# Editorial: Ecoepigenetics in Clonal and Inbreeding Plants: Transgenerational Adaptation and Environmental Variation

**DOI:** 10.3389/fpls.2019.00622

**Published:** 2019-05-15

**Authors:** Bi-Cheng Dong, Fei-Hai Yu, Sergio R. Roiloa

**Affiliations:** ^1^School of Nature Conservation, Beijing Forestry University, Beijing, China; ^2^Institute of Wetland Ecology & Clone Ecology / Zhejiang Provincial Key Laboratory of Plant Evolutionary Ecology and Conservation, Taizhou University, Taizhou, China; ^3^BioCost Group, Department of Biology, Faculty of Science, University of A Coruña, A Coruña, Spain

**Keywords:** clonal growth, environmental change, epigenetics, inbreeding plants, maternal effect, transgenerational plasticity

Accelerating global and regional environmental changes are likely to favor species that can rapidly adapt to new conditions. Long-lived, clonal species whose reproduction is mainly asexual have long been thought to possess a relatively low potential for adaptation. However, the potential for transmitting responses to environmental change between vegetative generations within clones could compensate for lack of natural selection based on sexual reproduction (Latzel and Klimešová, 2010; Douhovnikoff and Dodd, 2015). There are two well-studied mechanisms that underlie transgenerational environmental effects in clonal plants. First, transgenerational environmental effects on clonal (vegetative) offspring may depend on the quality of provisioning, similarly to seeds (Herman and Sultan, 2011; Dong et al., 2018). The relatively large size of clonal offspring may allow for more extensive provisioning with, e.g., carbohydrates or mineral nutrients, thereby obtaining greater fitness. Second, epigenetic changes may encode phenotypic plasticity and allow it to persist between vegetative generations (Dodd and Douhovnikoff, 2016; Richards et al., 2017). Changes such as DNA methylation, chromosome inactivation, and modifications of histones, chromatin, and small non-coding RNAs are now understood to transmit major phenotypic shifts between generations even in the absence of genetically based natural selection. This research topic assembles articles that deal explicitly with the ecological and evolutionary significance of transgenerational environmental effects in clonal plants, and that advance the understanding of the mechanisms of transgenerational effects in clones or inbreeding plants.

Three papers focus on the ecological significance of epigenetic regulation responses for clonal plants to different natural habitats. In a forum paper, Thiebaut et al. proposed how epigenetic regulation such as DNA methylation could cause chromatin dynamics and silencing, and influenced plant phenotypes, contributing to the adaptation of native plants, in the context of environmental variation. Broeck et al. showed the relationship between variability of DNA methylation and bud set phenology of the Lombardy poplar (*Populus nigra* cv. *Italica* Duroi) that is widely introduced in Europe. They suggest that epigenetic-based transgenerational inheritance may be relevant for adaption and evolution of *P. nigra* clones in contrasting or rapidly changing environments. Shi et al. reported that invasive populations of *Alternanthera philoxeroides* in China exhibit extremely low variation in DNA sequence, but high epigenetic diversity. They suggest that epigenetic variation may compensate for the loss of genetic variation in this invasive species and thus contribute to their success in novel environments.

Three papers report parental environmental effects on offspring fitness of clonal plants. Dong et al. tested effects of parental soil nutrient environments on offspring performance of the highly invasive, clonal herb *A. philoxeroides* at both the individual ramet level and the level of the whole generation of ramets. They provide novel evidence that the magnitude of parental environmental effects varied at different plant levels, and depended on propagule provisioning. Li et al. examined effects of parental shade environments on growth, morphological and physiological traits of a stoloniferous herb *Centella asiatica*. They found that transgenerational plasticity through both morphological and physiological flexibility was triggered across clonal generations of *C*. *asiatica* subjected to high/low light treatments, and such effects allowed offspring ramets to present adaptive phenotypes in response to the prevailing light environments. Fan et al. showed that physiological connection with parental ramets of a desert clonal shrub *Calligonum mongolicum* in favorable conditions can alleviate stress on offspring ramets exposed to wind erosion.

Two papers consider the variation in transgenerational environmental effects among genotypes. González et al. examined the generality of transgenerational environmental effects in the clonal plant *Trifolium repens* with five genotypes and five types of parental environments. They found that transgenerational environmental effects were highly genotype-specific and common in some genotypes, and potentially under epigenetic control. Baker et al. set up two glasshouse shade environments for an inbreeding plant *Polygonum persicaria*, and measured ecological important traits of their isogenic offspring in both environments. They found that the adaptive effects of parental shading were pronounced and highly significant for seedlings growing under shade, and such effects were mediated by DNA methylation status of parent plants, rather than changes to propagule provisioning.

Transgenerational environmental effects in sexually reproduced species have received considerable attention, but such effects in clonal plants have begun to attract interest. Researchers are recently attempting to advance understanding of the mechanisms for transgenerational environmental effects between vegetative generations, in the context of environmental variation. Clonal plants are widely distributed in nature and dominate a number of plant communities and ecosystems around the world. Therefore, knowledge of transgenerational environmental effects is important to understand how clonal plants can adapt efficiently to the ongoing, rapid change at both global and regional scales in natural environments. We hope the publication of this research topic will stimulate more studies on this important issue in the coming years.

## Author Contributions

All authors listed have made a substantial, direct and intellectual contribution to the work, and approved it for publication.

### Conflict of Interest Statement

The authors declare that the research was conducted in the absence of any commercial or financial relationships that could be construed as a potential conflict of interest.
